# Old Drugs and New Challenges: A Narrative Review of Nitazenes

**DOI:** 10.7759/cureus.40736

**Published:** 2023-06-21

**Authors:** Joseph Pergolizzi Jr, Robert Raffa, Jo Ann K LeQuang, Frank Breve, Giustino Varrassi

**Affiliations:** 1 Anesthesiology, Nema Research, Inc, Naples, USA; 2 School of Pharmacy, Temple University (Emeritus), Philadelphia, USA; 3 Pain Management, NEMA Research, Inc., Naples, USA; 4 Pharmacy, Temple University, Philadelphia, USA; 5 Pain Medicine, Paolo Procacci Foundation, Rome, ITA

**Keywords:** designer drugs, addiction, opioid use disorder, opioids, street drugs, nitazenes

## Abstract

Nitazenes are a group of compounds developed in the 1950s as opioid analgesics, but they were never approved to market. As such, they are not well known outside of academic research laboratories. A characteristic of nitazenes is their high potency (e.g., hundreds to thousands fold more potent than morphine and other opioids and tenfold more potent than fentanyl). In the past few years, several nitazenes, including “designer analogs,” have been detected in the illicit drug supply and have been implicated in overdose mortality, primarily due to their exceptionally high potency. In the street drug supply, nitazenes are often found mixed with fentanyl or other agents but their presence is not always disclosed to drug buyers, who may not even be familiar with nitazenes. These drugs pose a particular challenge since there is little experience in how to reverse a nitazene overdose or potential drug-drug or drug-alcohol interactions. Public health efforts are needed to better inform street drug consumers, first responders, healthcare professionals, and the general public about these “new old drugs” that are infiltrating the recreational drug supply.

## Introduction and background

In the late 1950s, the synthesis of 2-benzylbenzimidazole opioids led to the creation of several compounds now known collectively as nitazenes - although they do not technically meet the current United States Adopted Name (USAN) definition of an “azene.” They were of particular interest because their chemical structures are distinct from the typical morphine-like phenanthrene motif and meperidine analogs like fentanyl. The nitazenes were intended to be developed as analgesics but they were never approved for any therapeutic purpose [1,2] Their potency and street appeal caused them to be compared frequently to fentanyl, although they are structurally unrelated. As the Drug Enforcement Administration (DEA) and the Food and Drug Administration (FDA) have been better able to identify and schedule numerous fentanyl analogs [3], it appears that chemists in clandestine labs have gone back through historical pharmacology research literature for early attempts at developing synthetic opioids [4]. Novel psychoactive substances (NPS), including “novel” synthetic opioid analogs such as the re-emergence of the older nitazene drugs, are considered the driver in the recent upward trends in overdose mortality in the United States [5]. Despite the fact that nitazenes have been identified in the illicit recreational drug supply, few clinicians are aware of them or their implications for emergency medicine.

NPS are defined as synthetic agents possessing actual or alleged morphine-like or other psychoactive pharmacological properties that are not currently regulated by national or international law. The murky legal status of these substances combined with their potent psychoactivity and relatively inexpensive manufacturing costs makes them valuable street drugs [6]. Drug laws are based on specific agents, so the rapid and consistent emergence of novel drugs that enter the street market every year poses challenges to law enforcement [7,8]. While the DEA has recently introduced scheduling that makes many nitazenes illegal, the creation of new analogs can circumvent those rulings [9]. The aim of this narrative review is to describe nitazene drugs and to discuss the implications these dangerous new substances may have on clinical practice and society at large.

## Review

Methods

This is a narrative review that used the PubMed and Google Scholar databases to search for peer-reviewed articles on nitazenes. The authors also consulted authoritative websites such as those run by the DEA, the Centers for Disease Control and Prevention (CDC), and government-run websites for late-breaking information on these agents. Bibliographies of articles were checked for relevant resources as well. There is a paucity of information on nitazenes.

Results

Benzimidazole and its derivatives are well-known fungicides and used to treat nematode and trematode infections in animals and humans. The best known of these agents is the antifungal carbendazim. Among the many other derivatives of benzimidazole are etonitazene and clonitazene, which have been observed to have morphine-like effects on the human central nervous system due to their relatively selective affinity for the µ-opioid receptor [10]. These and similar analog agents were developed as analgesic drug candidates, but none was ever cleared for market release [11].

**Figure 1:**
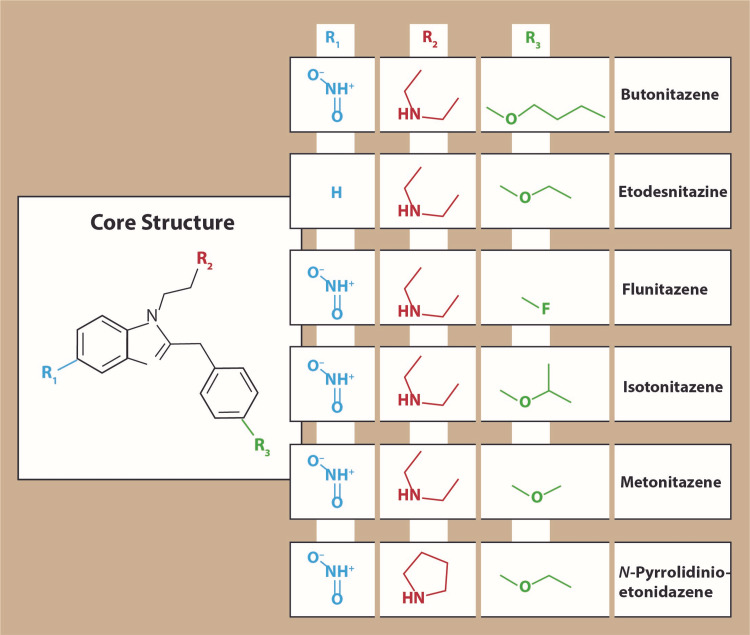
Several of the nitazene analogs recently detected in the street drug market. Of these agents, isotonitazene may be the best-known “street nitazene.” It is sometimes reported to be more potent than fentanyl.

The commercial failure of nitazenes has resulted in general pharmaceutical disinterest in these agents, but they remained of academic interest as tool drugs for research on opioid mechanisms of action and pharmacology, particularly etonitazene. In fact, nitazenes were nearly forgotten outside the specialized opioid research community until their sudden emergence in the past few years onto the illicit street market renewed interest in these synthetic opioids. Nitazenes are µ-opioid receptor agonists with psychoactive effects comparable to those of other µ-opioid receptor agonists, such as morphine, oxycodone, heroin, and so on [6]. The best known of the street agents is isotonitazene (street names “Iso” and “Tony”), a 5-nitro-2-benzylbenzimidazole opioid that was identified among street drugs in Europe in 2019 [12]. Structurally, isotonitazene is an analog of etonitazene, one of earliest nitazenes [11]. In the first half of 2020, metonitazene was identified in the recreational drug market [13]. To date, these drugs seem most prevalent in Europe and North America. The nitazene family consists of numerous analogs with the potential for more analogs to be added. The chemical structures of some of the main known nitazenes appear in Figure 1.

There is scant information on the pharmacokinetics and pharmacodynamics of the street nitazenes [14]. It has been speculated that these agents have a different orientation at the µ-opioid receptor compared to morphine or similar drugs [1]. Some, but not all, of nitazenes are more potent than fentanyl but there are limited and sometimes contradictory reports on this. Based on a systematic literature search of peripheral serum concentrations of isotonitazene from 93 fatalities, the potency of the isotonitazene is similar to that of fentanyl [6].

Isotonitazene has three metabolites that are active at the µ-opioid receptors: N-desethyl isotonitazene, 4’-OH-nitazene, and 5-aminoisotinazene.1 N-desethyl isotonitazene is more potent than the parent drug, but the N-desethyl metabolite of etonitazene is less potent than the parent drug [1].

Entry into the illicit drug supply

During the 1950s, nitazenes were developed by commercial pharmaceutical companies as synthetic opioid candidates and they were described in medical and pharmaceutical literature of the era. Thus, clandestine labs needed only to turn to the historic pharmacological literature to learn about the nitazene family. The European Monitoring Center for Drugs and Drug Addiction was first notified about the presence of isotonitazene in a biological sample obtained in July 2019 [15]. Since that time, isotonitazene has been implicated in over 200 drug-related overdose deaths in Europe and North America [7], but its presence likely is under-detected because many testing facilities are not set up to test for isotonitazene, or any other nitazenes, for that matter [8].

Metonitazene was first identified in the street drug supply during the COVID-19 pandemic (early 2020) [13]. Metonitazene has been confirmed in 20 authentic forensic autopsies with an average serum concentration level of 6.3 ± 7.5 ng/mL (median 3.8 ng/mL, range 0.5 to 33 ng/mL) and urine concentrations of 15 ± 13 ng/mL (median 11 ng/mL, range 6-46 ng/mL) [13]. In those 20 cases, metonitazene was the sole opioid found in 30% of the decedents, but metonitazene was more often used in combination with other drugs such as fentanyl, benzodiazepines, hallucinogens, and other opioids. Medical examiners listed metonitazene as the drug contributing to the death and the manner of death was determined to be accidental in all cases [13]. A 2021 analysis of unintentional drug overdose deaths that occurred in Knox County, Tennessee, United States, found that 26 of the 218 overdoses (12%) involved metonitazene combined with fentanyl [2].

Nitazenes are available in powders, counterfeit tablets, or liquids and may be mixed with inert substances and/or combined with other drugs, such as heroin, fentanyl, and benzodiazepines [7]. Their inclusion in other drug products is not detectable by consumers and may not be disclosed to them by sellers. When the new clandestine nitazenes entered the illicit market, they were not scheduled as controlled substances. In December of 2021, the DEA temporarily put numerous nitazenes on its Schedule I [9]. Since there are few validated methods to search for these substances and little is known about the network of clandestine labs and chemists who manufacture these drugs, the geographical distribution of these drugs is not known [7].

Clinical implications

The arrival of any new drug or intoxicant into the illicit drug supply creates tremendous burdens for first responders and emergency healthcare providers as they encounter the first overdose cases without the benefit of knowing much about the pharmacology, toxicology, or potential use or interactions of these novel agents. The problem is compounded by the fact that many street drug users may not be informed or aware of exactly what substances they have taken. Case in point: nitazenes were recently identified in batches of counterfeit hydromorphone tablets which were sold as pure, pharmacy-grade hydromorphone products [7]. Thus, the arrival of nitazenes to the street market exposes important knowledge gaps in the knowledge of drug users and their treatment providers. Clinicians, particularly emergency medicine professionals and first responders, need to know more about recreational street drugs in order to better treat overdose; other healthcare professionals and researchers need to know how to manage these specific drug use disorders, withdrawal, and rehabilitation; epidemiologists and public health experts need to know more to monitor drug trends.

Since nitazenes and other NPS are specifically developed to be cheap, easy to manufacture, highly intoxicating, and fall outside the official Controlled Substances Act (CSA), there are no quality, purity, or manufacturing standards. Even if street drug buyers understand they are buying heroin mixed with nitazenes, the quality and quantity of the nitazene would likely not be disclosed. The purveyors of these illicit street drugs may themselves be unaware of nitazene content in their products. The street drug market pattern with NPS is that as soon as an agent or group of agents is scheduled under the CSA (making them illegal), new NPS agents appear to take their place. Every new agent or derivatives bring challenges of identification, toxicity risk, and overdose treatment. The recent sharp increase in overdose mortality in the United States has been attributed to this trend toward NPS [6].

The challenges to clinical care are enormous. Patients in overdose-induced respiratory distress are often unresponsive and thus can provide no information to those treating them. Even if responsive or accompanied by other people, these overdose victims may not know what they have taken. Current drug testing equipment for nitazenes is often not available and conventional fentanyl test strips cannot detect their presence [16]. Thus, most clinicians treating people with drug-induced respiratory depression may not even consider the presence of nitazenes which, by dint of their potency, can complicate rescue.

In theory, some nitazenes can be antagonized by the opioid receptor antagonists, such as naloxone and nalmefene [6], but it is not clear if the high potency and impurities of the nitazenes might limit the effectiveness of the available antagonists. The lethal doses for nitazenes in humans, particularly in combination with other drugs or medical conditions, are not known. Many factors influence overdose-related morbidity and mortality, including not only the drug, but also the amount taken, the route of administration, possible drug-drug interactions or drug-alcohol interactions, body weight, opioid tolerance, and underlying health status.

Polysubstance abuse is increasingly prevalent [17], and the use of multiple drugs and/or alcohol concomitantly can result in a synergistic respiratory depression, with consequent decrease in oxygen saturation in the body, culminating in cerebral hypoxia [18,19]. While overdose toxicity is typically described as being either fatal or nonfatal with the implication that nonfatal overdoses cause no harm, opioid overdose is better thought of as occurring on a spectrum. Opioid overdose survivors may be left with short-term or long-term mental impairment or physical disabilities [18]. The neuropsychiatric term “toxic brain injury” has recently entered the medical lexicon to describe the increasingly encountered morbid results of a non-fatal opioid overdose [20]. The full implications of nitazenes on the trend and extent of opioid-overdose morbidity are difficult to estimate.

Public health implications

Nitazenes are not widely researched or discussed, even by authoritative agencies. A search on the Centers for Disease Control and Prevention (CDC) website on June 9, 2022 for the keyword “nitazene” yielded zero results, although these compounds were sometimes mentioned tangentially in articles about fentanyl. Clinicians, healthcare professionals, academics, public health officials, and policymakers may not be aware of nitazenes, the increasing presence of nitazenes in the illicit drug market, and the potential dangers of these powerful synthetic opioids. A broad-based public health campaign aimed at helping those who buy street drugs is crucial to help those who take recreational street drugs realize that nitazenes are a real, albeit currently invisible, threat. Such efforts could start with agencies like the CDC, DEA, and others, but could also be part of harm-reduction programs. Many of those who take illicit drugs have not even heard of nitazenes, much less are they aware of their risks. Even those who may be concerned about nitazenes in the street drug supply have no way to lab-test or otherwise identify nitazenes in recreational drugs.

Clinicians must work with specialized experts to develop protocols to treat confirmed or suspected nitazene overdose. These designer nitazene drugs may be considered esoteric drugs that evade conventional toxicology screens. First responders, emergency department clinicians, and others on the front lines must be informed that nitazenes have invaded the street drug market, that polysubstance drug abusers may be taking nitazenes without knowing it, and that nitazenes by themselves may be sold in counterfeit pills falsely labeled as popular pharmaceutical opioids.

As we better elucidate the pharmacokinetics and pharmacodynamics of these “old new” agents, rescue protocols should be developed to allow for the maximal survivability of a nitazene overdose. The abuse potential of these agents must be assessed and they must be integrated into protocols for drug testing and drug rehabilitation. It is not known if and to what extent the reversal or abrupt cessation of nitazenes may precipitate withdrawal symptoms, although withdrawal should be anticipated based on their receptor pharmacology. All of these questions urgently demand answers as current and future nitazenes are likely to be part of the illicit drug supply for the indefinite future.

Discussion

The costs of the opioid public health crisis are almost beyond measurement. In the fiscal year 2017-2018, it was estimated that the United States spent nearly $2 billion on treating opioid overdoses, of which $632 million went to emergency services alone [21]. When first responders can treat an overdose with no hospitalization, costs are around $3,000 per event [21 ]. Many rescued overdose patients refuse hospitalization, but of those who are hospitalized, 40% require intensive care [21]. Considering all of these factors, the United States spends about $11 billion annually on opioid overdoses, but this fails to account for lost productivity, associated costs for theft, law enforcement, criminal justice, homelessness, other healthcare services for drug-related conditions (infections, hepatitis), and rehabilitation programs, not to mention the costs of broken families, foster care, disrupted careers, and mental health services. Accounting for these, the costs go up to a staggering amount of more than $1 trillion a year [22]. Yet despite these expenditures, more Americans are dying of opioid overdose than at any other time in American history [23]. Overdose deaths are the main cause of death for Americans between the ages of 25 and 64 years [24] and most of the 93,000 annual deaths from drug overdose involve at least one opioid [25]. For every opioid-overdose fatality, there are 6.4 to 8.4 “nonfatal overdoses” which can be a source of long-term morbidity [18]. In fact, the prevalence and impact of non-fatal opioid overdose morbidity are likely underestimated.

Two themes emerge from our narrative review. The first is that nitazenes are likely going to continue to be part of the illicit drug market for the indefinite future. It is likely that more such compounds will be developed, particularly as some existing ones have been scheduled as controlled substances, thus becoming more risky to sellers. The National Forensic Laboratory Information System (NFLIS) has received 1,660 reports of nitazenes since 2019 [26]. They are most frequently combined with fentanyl, but may also be used with benzodiazepines, cocaine, heroin, methamphetamine, and tramadol. It seems that nitazenes are here to stay.

The second, more alarming, theme is that the model of repurposing an old psychoactive drug or spinning off analogs to psychoactive agents does not exceed the abilities of the many chemists working in the burgeoning clandestine laboratories around the world. They will likely persist in introducing new and potentially deadly substances to the street drug market as long as it is possible and profitable. Steps should be taken to “harden” countries against such agents infiltrating illegal drug markets and preparations made to deal with associated overdoses. These steps may involve early and efficient ways to detect these drugs along with educational campaigns to alert street users and healthcare providers. Most importantly, serious efforts and not lip service need to be directed to the psychosocial causes and understanding of substance use disorders and their humane, informed, and evidence-based treatment.

## Conclusions

Certain nitazenes can be more potent than fentanyl and are increasingly found on the illicit drug market in North America and Europe. These agents may not be disclosed to buyers of illicit drugs and are often mixed with fentanyl or other agents. They are associated with morbidity and mortality, and it is not known how effective naloxone or other antagonists are when reversing nitazene-induced respiratory depression. The healthcare system needs to be better educated about these agents and public health efforts are urgently required to better educate policymakers, healthcare professionals, clinicians, and the public about the existence and dangers of these drugs.
